# Multi-omics analysis reveals the landscape of tumor microenvironments in left-sided and right-sided colon cancer

**DOI:** 10.3389/fmed.2024.1403171

**Published:** 2024-08-29

**Authors:** Dongfang Liu, Chen Li, Zenghua Deng, Nan Luo, Wenxia Li, Wenzhe Hu, Xiang Li, Zichao Qiu, Jianfei Chen, Jirun Peng

**Affiliations:** ^1^Department of Surgery, Beijing Shijitan Hospital, Capital Medical University, Beijing, China; ^2^Ninth School of Clinical Medicine, Peking University, Beijing, China

**Keywords:** TME, colorectal cancer, right-sided colon cancer, left-sided colon cancer, immune therapy, PD-1

## Abstract

**Background:**

Distinct clinical features and molecular characteristics of left-sided colon cancer (LCC) and right-sided colon cancer (RCC) suggest significant variations in their tumor microenvironments (TME). These differences can impact the efficacy of immunotherapy, making it essential to investigate and understand these disparities.

**Methods:**

We conducted a multi-omics analysis, including bulk RNA sequencing (bulk RNA-seq), single-cell RNA sequencing (scRNA-seq), and whole-exome sequencing (WES), to investigate the constituents and characteristic differences of the tumor microenvironment (TME) in left-sided colon cancer (LCC) and right-sided colon cancer (RCC).

**Result:**

Deconvolution algorithms revealed significant differences in infiltrated immune cells between left-sided colon cancer (LCC) and right-sided colon cancer (RCC), including dendritic cells, neutrophils, natural killer (NK) cells, CD4 and CD8 T cells, and M1 macrophages (P < 0.05). Notably, whole-exome sequencing (WES) data analysis showed a significantly higher mutation frequency in RCC compared to LCC (82,187/162 versus 18,726/115, *P* < 0.01). Single-cell analysis identified predominant tumor cell subclusters in RCC characterized by heightened proliferative potential and increased expression of major histocompatibility complex class I molecules. However, the main CD8 + T cell subpopulations in RCC exhibited a highly differentiated state, marked by T cell exhaustion and recent activation, defined as tumor-specific cytotoxic T lymphocytes (CTLs). Immunofluorescence and flow cytometry results confirmed this trend. Additionally, intercellular communication analysis demonstrated a greater quantity and intensity of interactions between tumor-specific CTLs and tumor cells in RCC.

**Conclusion:**

RCC patients with an abundance of tumor-specific cytotoxic T lymphocytes (CTLs) and increased immunogenicity of tumor cells in the TME may be better candidates for immune checkpoint inhibitor therapy.

## 1 Introduction

Colorectal cancer (CRC) is the most common malignant tumor in the digestive system and the third most prevalent cancer worldwide. Additionally, it is the second leading cause of cancer-related deaths (1). The established treatments for colorectal cancer include surgery, radiation therapy, chemotherapy, and targeted therapy. Despite significant advancements and favorable outcomes for early-stage patients, these interventions are less effective for advanced-stage patients.

Colon cancer can be classified based on the tumor’s location into right-sided colon cancer (RCC) and left-sided colon cancer (LCC). RCC includes cancers of the cecum, ascending colon, and hepatic flexure, while LCC includes cancers of the splenic flexure, descending colon, and sigmoid colon. These different anatomical locations are associated with distinct clinical manifestations and molecular characteristics (1–3) 1. Previous studies have shown that patients with left-sided colon cancer (LCC) are more responsive to chemotherapy and EGFR monoclonal antibody therapy, whereas patients with right-sided colon cancer (RCC) have limited responses to these treatments (4). In recent years, immunotherapy with immune checkpoint inhibitors (such as anti-PD-1/PD-L1, CTLA-4, and LAG3 monoclonal antibodies) has achieved significant breakthroughs in treating advanced tumors and shown remarkable therapeutic effects in multiple cancer types (5, 6). However, despite the promising efficacy of immunotherapy in many tumors, a significant proportion of patients do not respond to these treatments (7). According to the latest NCCN guidelines, advanced-stage CRC patients with dMMR/MSI-H phenotypes are recommended for anti-PD-1/PD-L1 treatment. However, only a small percentage of CRC patients (around 5–8%) have dMMR/MSI-H mutations, limiting the potential benefits of immunotherapy for the broader CRC patient population (8). It is essential to identify new molecular subtypes for the remaining patients to better evaluate their response to immunotherapy.

The tumor microenvironment (TME) significantly affects the response to immunotherapy and prognosis in cancer patients (9). The TME is a complex mixture of cells, including tumor cells, stromal cells, immune cells, vascular cells, and extracellular matrix cells. Previous studies have shown that an increased presence of plasma cells, dendritic cells, mast cells, and activated memory CD4 + T cells, along with a decreased presence of M0, M1, and M2 macrophages, is linked to a poor prognosis in colon cancer (10). The molecular phenotypic variations in different regions of colon cancer may contribute to differences in the composition and phenotype of cells within the TME between left-sided colon cancer (LCC) and right-sided colon cancer (RCC). Additionally, prior research indicates that myeloid-derived suppressor cells (MDSCs) are more prevalent in the TME of RCC patients compared to LCC patients. The increased presence of MDSCs in the TME is associated with an unfavorable prognosis for colon cancer patients (2). Despite these findings, there is limited scholarly literature on the comprehensive investigation of the TME in different locations of colon cancer using a multi-omics approach. To address this gap, the current study aims to employ various methodologies, including single-cell RNA sequencing, bulk RNA sequencing, whole exome sequencing, immunohistochemistry, and flow cytometry, to thoroughly explore and elucidate the complexities of the TME in LCC and RCC.

## 2 Materials and methods

### 2.1 Data sources and processing

Bulk RNA-seq data, clinical information, and SNP mutation site data for colon cancers were obtained from the TCGA database (https://portal.gdc.cancer.gov/). This dataset includes 59 normal tissue samples and 453 colorectal adenocarcinoma (COAD) samples. Samples lacking complete survival information, location details, and other pertinent clinical data were excluded, resulting in a refined training set of 312 COAD patients for this study. Additionally, the GSE103479 dataset, containing 122 COAD patients with comprehensive survival and location information, was downloaded from the GEO database to validate the model’s feasibility. Patient information is detailed in Supplementary Table 1. Furthermore, the CRC scRNA-seq dataset GSE200997, also from the GEO database, includes 16 samples of primary tumors and 8 corresponding adjacent normal tissue samples. Samples were integrated using the anchors method within the R package “Seurat” (11). Core cells were identified by filtering the scRNA-seq data. Cells ineligible for analysis, including those with genes detectable in three or fewer cells and low-quality cells with fewer than 200 detected genes, were excluded. Dimensionality reduction analysis was performed using the Uniform Manifold Approximation and Projection (UMAP) algorithm for a comprehensive assessment.

### 2.2 Major cell type identification and data visualization

Using the Seurat FindAllMarkers function, we assessed the differentially expressed markers for each cell group. Genes with an average expression in a subcluster that was log2-fold higher than in other subclusters were identified. We used marker genes with the highest fold expression within each cluster for this analysis. Additionally, to identify cell types, we utilized the SingleR package (12) and extensive transcriptomic datasets that include well-annotated cell types.

### 2.3 Trajectory analysis

We used a reverse graph embedding approach with Monocle2 to reconstruct single-cell trajectories within major cell types (13). We created a CellDataSet object using UMI count matrices and the negbinomial.size () function with default settings. Cells were grouped and projected onto t-SNE. To measure the average transcriptional transition a cell undergoes from one state to another, we quantified the cumulative duration of the trajectory. Additionally, we conducted trajectory analysis with the Slingshot R package, which uses minimum spanning trees to map multiple branching lineages. The snapshot wrapper function was used to integrate UMAP dimensionality reduction and cluster labels, consistent with Seurat objects. This combined approach improved the robustness and comprehensiveness of single-cell trajectory reconstruction across major cell type.

### 2.4 Analysis of immune cells infiltration score and immunotherapy response score

We used several deconvolution algorithms—TIMER, CIBERSORT, QUANTISEQ, XCELL, MCPCOUNTER, and EPIC—to estimate immune cell infiltration in tumor tissues, based on their bulk RNA-Seq gene expression profiles (14). We assessed significance using the purity-adjusted Spearman rank correlation test, which provided P values and partial correlation values. The results were visually represented with a heatmap and a box plot to clearly illustrate the immune landscape within the tumor microenvironment. Additionally, we used the Immunophenoscore (IPS) to predict patient responses to immune checkpoint inhibitors, such as PD-1 and CTLA-4, in the TCGA database. The IPS integrates indicators like immune checkpoint expression levels, MHC expression levels, and suppressive immune cell levels. This score is available from the TCIA database (https://tcia.at/patients) (15).

### 2.5 Intercellular communication analysis

We conducted the intercellular communication analysis using the R package CellChat (16). For the intercellular communication analysis, T cells and tumor cells were categorized into subgroups. We began by creating a CellChat object with the ‘createCellChat’ function. After annotating this object and identifying overexpressed genes, we calculated communication probabilities using the ‘computeCommunProb’ function. We then detailed the communications of each cell signaling pathway with the ‘compute_Commun_ProbPathway’ function. Finally, we visualized these communications using the ‘netVisual_chord_gene’ function.

### 2.6 Analysis of somatic mutations

To assess the mutational burden in colorectal cancer (COAD), we used the R package TCGAbiolinks to retrieve mutation data. We then analyzed this data with the maftools package (17) to determine the Tumor Mutational Burden (TMB) and assess differences in TMB within the study context.

### 2.7 Clinical samples

The study adhered to the ethical guidelines of the 1975 Declaration of Helsinki and the regulations set by the National Natural Science Foundation of China. Approval was granted by the Ethical Committee of Beijing Shijitan Hospital. Clinical samples were collected from June 2022 to June 2023 at Beijing Shijitan Hospital, Capital Medical University, with informed consent obtained from patients undergoing surgery. A total of 12 clinical samples were collected, including 6 from left-sided colon cancer (LCC) and 6 from right-sided colon cancer (RCC). Clinical details of the patients are provided in Supplementary Table 2.

### 2.8 Immunofluorescence

Tissue sections were deparaffinized in xylene and rehydrated through a series of graded ethanol solutions. Antigen retrieval was performed using a citrate buffer (pH 6) with heat. The fixed tissue samples were washed with PBS and blocked with 5% BSA for 2 hours. Primary antibodies, diluted in antibody buffer, were incubated with the tissues overnight. The following day, tissues were washed with PBS and incubated with fluorochrome-conjugated secondary antibodies. After another round of washing, tissues were mounted with Antifade Mounting Medium containing DAPI and allowed to dry. Images were captured using a Nikon confocal microscopy system. The antibodies used are listed in Supplementary Table 3.

### 2.9 Tissue digestion and cell preparation

Tumor tissues were cut into approximately 0.5 mm^3^ pieces and digested in 6 mL RPMI medium containing 0.5 mg/mL collagenase type IV (Sigma Aldrich) and 0.05 mg/mL DNAse I (Roche) for 10 min at 37°C with shaking at 300 rpm. The samples were then homogenized by passing through a 70 μm filter (BD Biosciences, Falcon, USA) and centrifuged for 10 min at 4°C and 1500 rpm. Tumor cells were further purified using 30% Percoll (Cytiva, USA) and centrifuged for 20 min at 500 × g at room temperature. The cell pellet was resuspended and washed with ice-cold PBS.

### 2.10 Flow cytometric analysis

Single cells were isolated from the tumor tissues as described. To block Fc receptors, FcR Blocking Reagent (Miltenyi Biotech) was added and incubated for 5–10 min at 4°C. Cells were then incubated with surface marker-specific antibodies for 30 min at 4°C. After washing twice with MACS buffer (0.5% bovine serum albumin in PBS), the cells were resuspended in MACS buffer and analyzed using a FACS Canto II flow cytometer (BD Biosciences). Data were processed with FlowJo software (Tree Star, OR, USA). Dead and live cells were differentiated using Ghost Dye (TONBO). The antibodies used are listed in Supplementary Table 3.

### 2.11 Statistical analysis

To obtain mean values and standard deviations, three independent experiments were performed. Multiple comparisons were assessed using one-way analysis of variance with Bonferroni’s *post-test*, while pairwise comparisons were conducted with Student’s *t*-tests. Pearson’s correlation test was used for correlation analyses. Statistical significance was defined as a *p*-value of less than 0.05.

## 3 Results

### 3.1 Differences in prognosis and tumor microenvironment between left-sided and right-sided colon cancer

**FIGURE 1:**
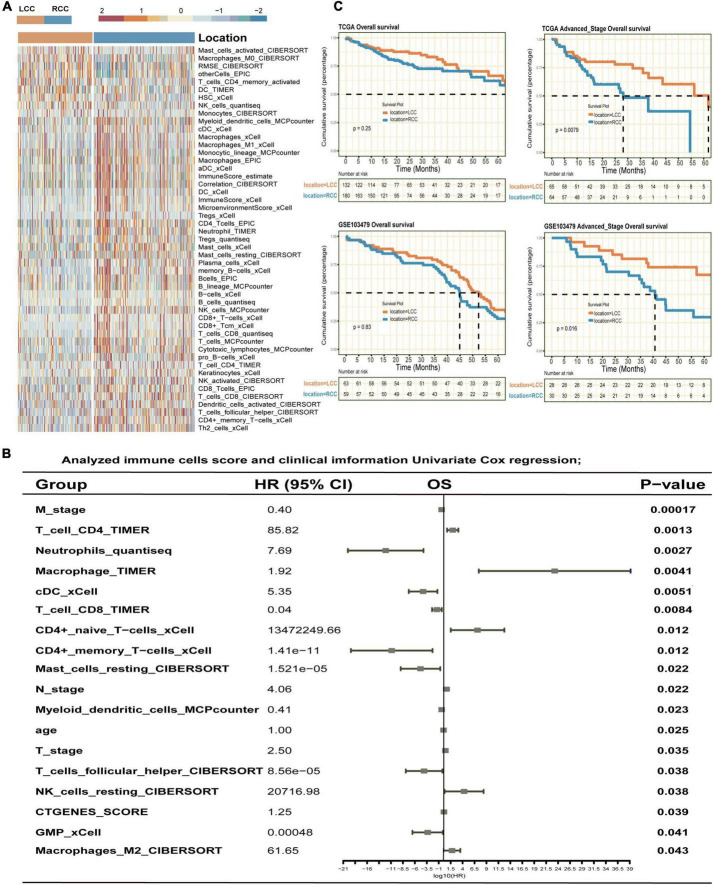
The immune landscape and prognosis differences between LCC and RCC of bulk RNA-seq datasets. **(A)** The immune infiltration heatmap of LCC and RCC. **(B)** Univariate Cox regression analysis of COAD immune infiltration score and clinical index. **(C)** Kaplan-Meier method was used to analyze the overall survival time of LCC and RCC samples from the TCGA and GSE103479 datasets.

We analyzed tumor microenvironment (TME) scores from TCGA and GEO databases using deconvolution algorithms (Supplementary Table 4). This analysis revealed significant differences in TME profiles between left-sided colon cancer (LCC) and right-sided colon cancer (RCC). Specifically, LCC showed higher scores for M0 macrophages, activated CD4 + memory T cells, dendritic cells (DC), natural killer (NK) cells, and monocytes. In contrast, RCC had higher scores for M1 macrophages, neutrophils, and CD8 + T cells (Figure 1A, Supplementary Figure 1). Univariate Cox regression analysis identified that infiltration by neutrophils, conventional dendritic cells (cDC), CD4 + memory T cells, mast cells, and T follicular helper cells was associated with a better prognosis in colon cancer. Conversely, infiltration by macrophages, CD4 + naïve T cells, and resting natural killer cells was linked to a poorer prognosis (Figure 1B). Additionally, we compared the prognoses of patients with LCC and RCC. Patients with LCC had a slightly better prognosis compared to those with RCC across all stages (Figure 1C). Notably, for advanced stage (III/IV) colon cancer, patients with LCC had a significantly better prognosis than those with RCC, as shown by the TCGA dataset (LCC vs RCC: 25.2 months vs 16.9 months, *P* = 0.0079) and the GEO dataset (LCC vs RCC: 49.3 months vs 39.0 months, *P* = 0.016).

### 3.2 Identifying cell clusters in colon cancer single-cell RNA-sequencing data reveals high heterogeneity in TME between LCC and RCC

**FIGURE 2:**
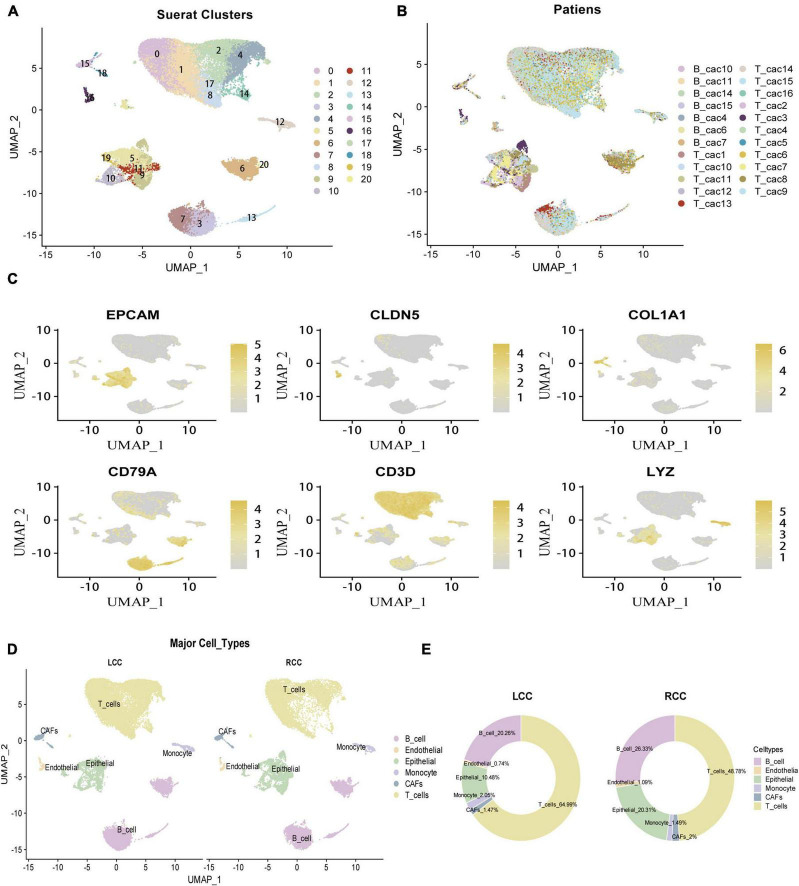
Identifcation of 6 cell clusters with diverse annotations revealing high cellular heterogeneity in COAD tumors based on single-cell RNA-seq Data. **(A)** The umap algorithm was applied to the top 20 PCs for dimensionality reduction, and 21 cell clusters were successfully classified. **(B)** Classifcation of cell clusters in each sample. **(C)** Identifcation of various cell types based on expression of specifed marker genes. **(D)** All 6 cell clusters in COAD were annotated with singleR and CellMarker according to the composition of marker genes. **(E)** The proportion of cell types in LCC and RCC.

To explore differences in the tumor microenvironment (TME) between left-sided colon cancer (LCC) and right-sided colon cancer (RCC), we analyzed single-cell RNA-sequencing (scRNA-seq) data from colon cancer cells across different anatomical locations. After rigorous quality control, we obtained 42,696 cells for further analysis (Supplementary Table 5). The data preprocessing results are detailed in Supplementary Figure 2. Following log normalization and dimensionality reduction, we identified 21 distinct cell clusters (Figure 2A), which were visualized across all samples (Figure 2B). Cells were classified into specific types based on canonical marker genes (Supplementary Table 6), including epithelial cells (EPCAM +), fibroblasts (COL1A1 +), endothelial cells (CLDN5 +), T cells (CD3D +), B cells (CD79A +), and monocytes (LYZ +) (Figure 2C). To assess the heterogeneity in the TME of LCC and RCC, we analyzed 26,124 cells from tumor tissues of 8 LCC and 8 RCC patients. The distribution and proportion of various cell types in different LCC and RCC tissues were examined (Figures 2D, E). Our results showed notable differences in the proportions of epithelial cells (tumor cells) and T cells, highlighting significant heterogeneity in the TME across different anatomical sites in colon cancer.

### 3.3 Tumor cells in RCC exhibit higher malignancy and immunogenicity

**FIGURE 3:**
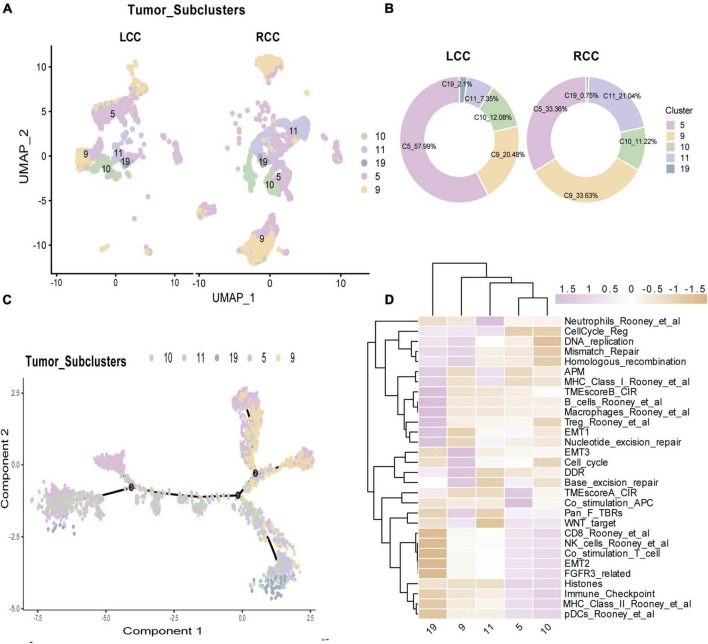
Cell proportions, Gene set enrichment and trajectories of tumor cells. **(A)** 5 tumor cell subpopulations in LCC and RCC. **(B)** The proportion of tumor cell subpopulations in LCC and RCC. **(C)** Trajectory analysis of tumor cell colored by subpopulations. **(D)** Gene set enrichment of 5 tumor cell subclusters.

The tumor microenvironment (TME) in solid tumors consists of complex components, with tumor cells being a principal factor influencing prognosis. The heterogeneity of tumor cells plays a crucial role in shaping cancer patients’ outcomes. To explore this heterogeneity in colon cancer, we analyzed tumor cell subpopulations across different anatomical locations. We isolated epithelial cells from tumor tissues and identified 4,632 tumor cells for further analysis. Using initial clustering results, we categorized these cells into five distinct tumor cell subpopulations (Figure 3A). We then compared the proportions of these subpopulations between LCC and RCC. In LCC, the predominant subpopulation was C5 (LCC vs RCC: 57.99% vs 33.36%), while in RCC, subpopulations C9 (LCC vs RCC: 20.40% vs 33.63%) and C11 (LCC vs RCC: 7.35% vs 21.04%) were more prevalent (Figure 3B). Next, we examined the differentiation trajectories of these subpopulations using Monocle. The analysis showed that subpopulations C5 and C10 exhibited high differentiation levels, indicating more mature epithelial tumor cells, whereas subpopulation C9 showed low differentiation, suggesting higher malignancy in RCC (Figure 3C). Additionally, we evaluated the functions of different tumor cell subpopulations using the GSVA algorithm. Our results indicated that the dominant C5 subpopulation in LCC had low expression of MHC I, which may suggest a deficiency in TCR-MHC interactions and potentially lead to a poor response to immunotherapy (18, 19). Conversely, the dominant C9 subpopulation in RCC exhibited characteristics of low differentiation, such as deficiencies in DNA mismatch repair, cell cycle regulation, and epithelial-mesenchymal transition. Another notable subpopulation in RCC, C11, showed strong cell proliferation and high expression of MHC I (Figure 3D), which suggests a potential for a favorable response to immune interventions.

### 3.4 Higher frequency of missense mutations in RCC suggests potentially greater immunogenicity

**FIGURE 4:**
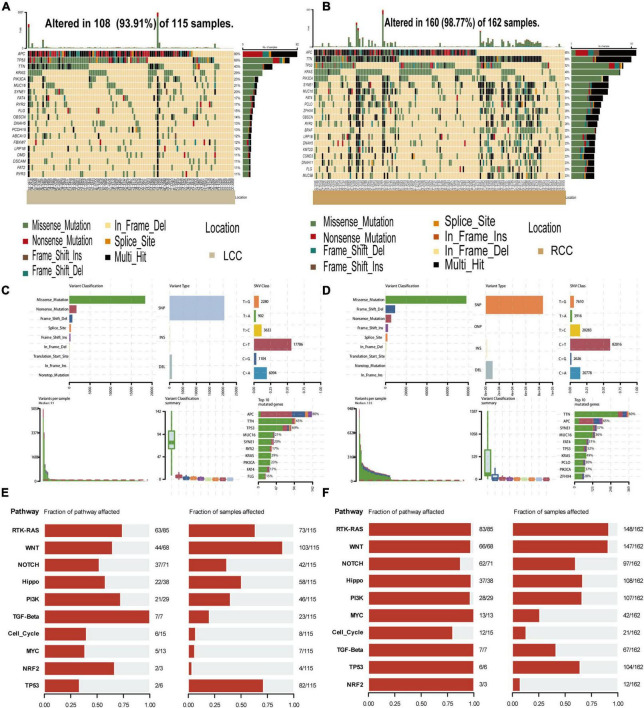
The mutations landscape analysis of LCC and RCC. **(A,B)** The tumor mutational burden (TMB) of of LCC and RCC. **(C,D)** Overall description of the LCC and RCC patient mutation landscape. **(E,F)** Functional analysis of the mutated genes in LCC and RCC.

Tumor mutation burden (TMB) is crucial for the effectiveness of immunotherapy. To investigate this, we analyzed somatic mutations in LCC and RCC patients using the maftools package. Our findings revealed that in colon cancer, the primary gene mutations involved APC, TTN, TP53, MUC16, SYNE1, RYR2, and KRAS, predominantly characterized by missense mutations and SNPs, with the most common mutation being the substitution of C with T. Notably, RCC exhibited a higher frequency of missense mutations and SNPs compared to LCC (missense mutations: LCC vs. RCC: 18726/115 vs. 82187/162; SNPs: LCC vs. RCC: 32524/115 vs. 144253/162) (Figures 4A, B, Supplementary Table 7). Functional analysis of these mutations showed that they primarily affected protumor growth and progression pathways (e.g., RTK-RAS, WNT, NOTCH, PI3K, MYC) (Figures 4E, F). Furthermore, the proportion of tumor development driven by these mutations was higher in RCC patients compared to those with LCC (Figures 4C, D). The greater number of missense mutations and SNPs in RCC suggests that these tumors are likely to produce more neoantigens, potentially leading to increased infiltration of tumor-specific cytotoxic T lymphocytes (CTLs) and a stronger immune response within the tumor microenvironment (20).

### 3.5 RCC exhibits higher infiltration of tumor-specific T cells

**FIGURE 5:**
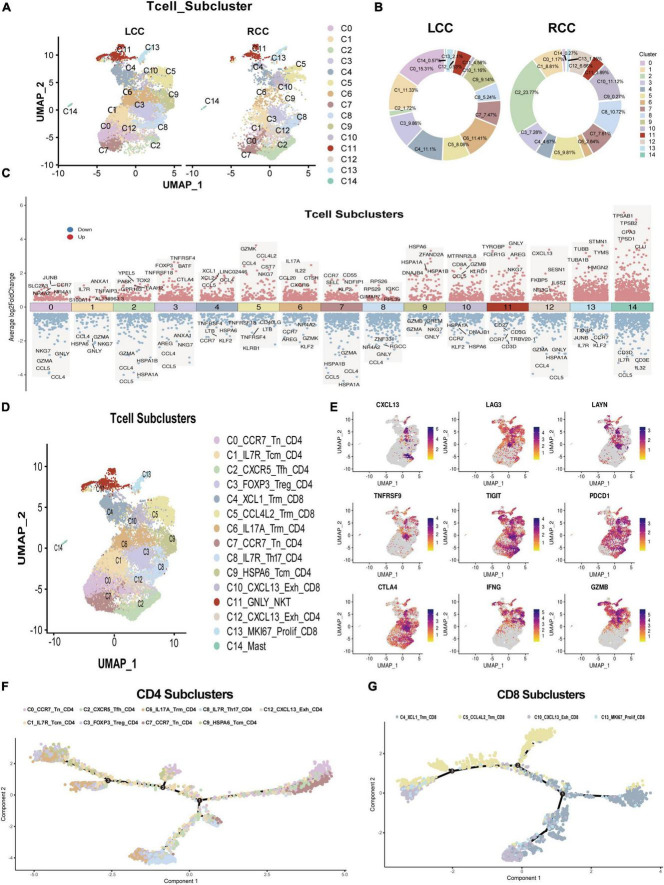
Single-cell seq revealed T cell feature difference between LCC and RCC. **(A)** After dimensionality reduction analysis, 15 T cell subpopulations obtained from LCC and RCC. **(B)** The proportion of T cell subpopulations in LCC and RCC. **(C)** Differential gene expression analysis shows up(red) and down(blue) regulated genes across all 15 subpopulations. **(D)** Annotation of 15 T cell subpopulations. **(E)** Distribution of T cell exhaustion and activation related molecules in T cell clusters. **(F)** Trajectory analysis of CD4 + T cell colored by subpopulations. **(G)** Trajectory analysis of CD8 + T cell colored by subpopulations.

To explore differences in T cell subsets between LCC and RCC, we analyzed 15,118 T cells from the dataset and performed dimensionality reduction. This analysis revealed 15 distinct T cell subclusters (Figure 5A). Comparing these subclusters between LCC and RCC, we found notable differences. Specifically, subclusters C0, C6, and C9 were more prevalent in LCC, while subclusters C2, C10, and C12 were more common in RCC tumors (Figure 5B). To further characterize these T cell subclusters, we conducted differential gene expression (DGE) analysis, which identified genes with varying expression levels across the T cell clusters (Figure 5C; Supplementary Table 8). We also performed single-cell gene set enrichment analysis (ScGSEA) to gain insights into the phenotypic profiles of tumor-infiltrating lymphocytes (TILs). This involved evaluating the expression of cluster-specific markers and analyzing over 100 gene signatures from recent single-cell RNA sequencing studies (Supplementary Table 9) (21–25). Among the identified T cell subclusters, CD4 T cells were mainly found in clusters C0, C1, C2, C3, C6, C7, C8, C9, and C12, while CD8 T cells were primarily located in clusters C4, C5, C10, and C13. CD4 T cells were further classified into several distinct subsets: naïve CD4 T cells (C0, C7), central memory CD4 T cells (C1, C8, C9), follicular helper CD4 T cells (C2), regulatory CD4 T cells (C3), Th17 CD4 T cells (C6), and exhausted CD4 T cells (C12). Similarly, CD8 T cells were categorized into tissue resident memory CD8 T cells (C4, C5), exhausted CD8 T cells (C10), and proliferating CD8 T cells (C13) (Figure 5D). Notably, the C10 cluster, predominant in RCC tumors, displayed characteristics of exhausted effector T cells. These cells showed increased expression of genes such as CXCL13, LAG3, LAYN, TNFRSF9, TIGIT, PDCD1, CTLA4, IFNG, and GZMB. We identified these as tumor-specific CTLs, consistent with findings from our previous studies (26, 27) (Figure 5E). The cell subpopulations identified are significant for the effectiveness of immune checkpoint therapies. Analysis of differentiation trajectories using the Monocle algorithm revealed that the C3_FOXP3_Treg_CD4 + and C10_CXCL13_Exh_CD8 + subsets represent terminally differentiated T cell subclusters (Figures 5F, G). These findings indicate that RCC tumors have a higher presence of tumor-specific CTLs compared to LCC tumors. Overall, this research highlights the distinct characteristics and phenotypes of T cell subclusters in the tumor microenvironment of LCC and RCC, offering valuable insights into the immune landscape of colon cancer.

### 3.6 Elevated PD1 expression in CD8 + T cells in RCC compared to CD4 + T cells in LCC

**FIGURE 6:**
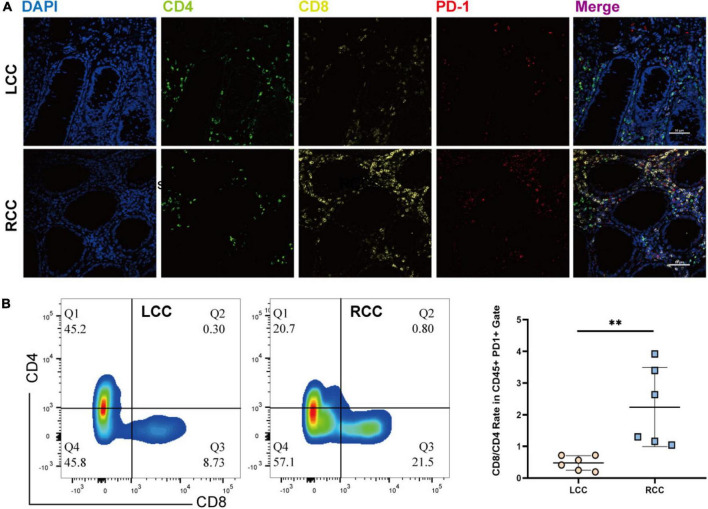
The immunofluorescence and Flow Cytometric examination of the infiltrating immune cell in tumors of LCC and RCC. **(A)** Immunofluorescence examinate CD4 (FITC, Green), CD8 (Cy5, Yellow), PD-1(Cy3, Red) protein expression in the TME of LCC and RCC. **(B)** Flow Cytometric examinate the frequency of PD1 + CD4 and PD1 + CD8 T-cell in the TME of LCC and RCC. **p < 0.01.

The frequency of PD1 expression on infiltrating lymphocytes is a key indicator of response to immune checkpoint inhibitors. We performed immunofluorescence staining on tumor samples from both LCC and RCC, using lymphocyte markers CD4 and CD8, along with the exhaustion marker PD1. The analysis revealed that RCC tumors had a higher proportion of CD8 T cells compared to LCC tumors. Gating strategy is shown in (Supplementary Figure 3). Specifically, the percentage of CD8 + PD1 + lymphocytes was greater in RCC patients (Figure 6A). Flow cytometry further confirmed these findings, showing that RCC patients had a higher proportion of CD8 + lymphocytes and a lower proportion of CD4 + T cells compared to LCC patients. In terms of PD1 + immune cells, CD4 + T cells were more prevalent in LCC patients (18.7–51.6%) compared to RCC (5.82–20.7%), while PD1 + CD8 + T cells were more common in RCC patients (22.1–22.8%) compared to LCC (8.73–18.29%) (Figure 6B, Supplementary Table 10). These results are consistent with the immunofluorescence findings, indicating that RCC tumors have a higher abundance of tumor-specific cytotoxic T lymphocytes (CTLs) and elevated PD1 expression. This suggests that RCC patients might respond better to immune checkpoint inhibitor treatments.

### 3.7 Higher frequency of lymphocyte-mediated tumor cell killing in RCC

**FIGURE 7:**
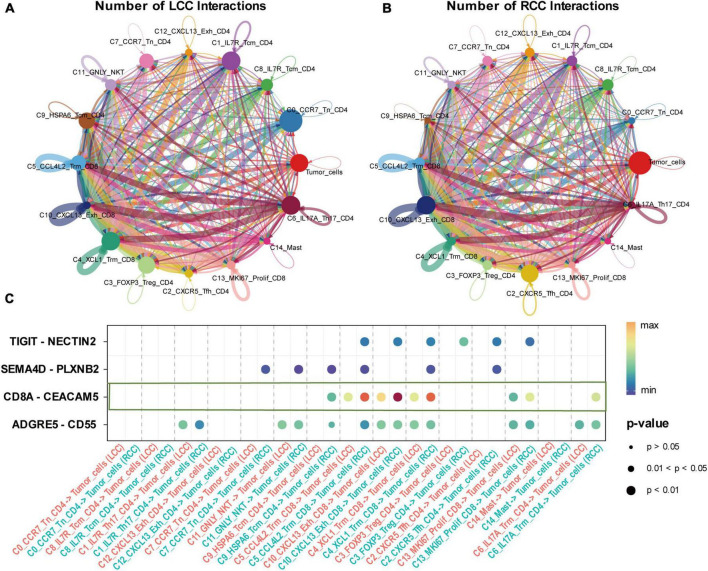
Interaction between T cell subpopulations and tumor cells of LCC and RCC. **(A,B)** The number of interactions between T cell subpopulations and tumor cells of LCC and RCC, the thickness of the connecting lines represents the quantity of mutual interactions. **(C)** The signaling pathways of the interaction between LCC and RCC, with the color depth of the bubbles representing the strength of the interaction and the size of the bubbles representing the *P*-value.

The effectiveness of cancer immunotherapy, especially checkpoint treatments, depends significantly on the presence and interaction of tumor-specific cytotoxic T lymphocytes (CTLs) within the tumor microenvironment. To explore how tumor cells interact with immune cells in LCC and RCC, we employed the CellChat algorithm for analysis. Our findings show that in LCC, there is close interaction between lymphocytes, particularly between initial cells and CD4 + cells. CD4 + FOXP3 + Treg cells also demonstrated extensive communication with other cells in LCC, but there was relatively limited interaction between immune cells and tumor cells. In contrast, RCC tumors exhibited more frequent and intense interactions between immune cells and tumor cells. Specific cell clusters, such as C2_CXCR5_Tem_CD4 and C10_CXCL13_Exh_CD8, showed extensive communication with other cells, indicating a more sophisticated immune response mechanism in RCC (Figures 7A, B). Analysis of communication pathways revealed key interactions including TIGIT−NECTIN2, SEMA4D−PLXNB2, CD8A−CEACAM5, and ADGRE5−CD55. The intensity of these interactions was significantly higher in RCC compared to LCC (Figure 7C).

### 3.8 RCC patients show higher responsiveness to immune checkpoint inhibitors

**FIGURE 8:**
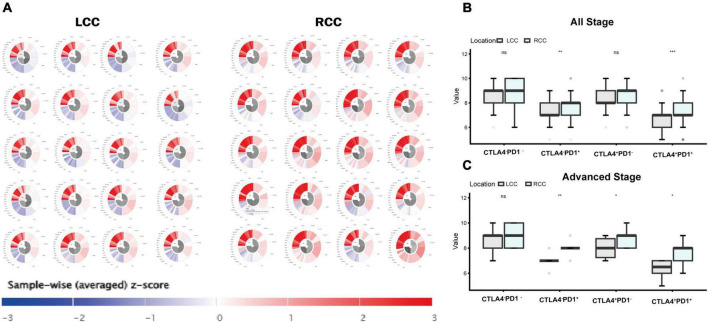
Immunophenoscores and Response to immune Checkpoint Blockade. **(A)** Presented are immunophenograms delineating individual patients with LCC or RCC, the top left quadrant represents Antigen Processing score, the bottom left quadrant represents Checkpoints Immunomodulators score, the top right quadrant represents Effector Cells score, and the bottom right quadrant represents Suppressor Cells score. The red color indicates a high score and blue represents low score. **(B)** IPS of response to blockade with anti-Checkpoint antibody of all stage LCC and RCC patients. **(C)** IPS of response to blockade with anti-Checkpoint antibody of advanced stage LCC and RCC patients.

We compared the Immune Prognostic Score (IPS) between LCC patients (*n* = 132) and RCC patients (*n* = 180) using TCGA datasets. The IPS, derived from bulk RNA-sequencing data, reflects various factors such as antigen processing, checkpoint immunomodulators, effector cells, and suppressor cells, to predict the efficacy of immune checkpoint inhibitors (Supplementary Figure 4) (28). We randomly selected 20 patients from each group and presented their predicted responses to immune checkpoint inhibitors (Figure 8A). Analysis of IPS scores for all patients revealed that RCC patients showed a significantly better response to these inhibitors (*P* < 0.05) (Figure 8B). This disparity was even more pronounced in advanced stage colon cancer, where RCC patients (*n* = 68) had a significantly better response compared to LCC patients (*n* = 67) (*P* < 0.01) (Figure 8C).

## 4 Discussion

Clinical trials have demonstrated the potential effectiveness of immunotherapy for advanced cancer; however, the benefits are limited for some patients due to variations in the immune microenvironment (29–31).

Most previous studies on immunotherapy for colon cancer have focused on the tumor’s microsatellite instability (MSI) status (32). There is, however, a lack of comprehensive research on how immunotherapy responses and immune microenvironments differ between colon cancer cases originating from different anatomical sites. To address this, our study combined single-cell RNA sequencing, bulk RNA sequencing, whole exome sequencing (WES), immunohistochemistry, and flow cytometry to explore differences in the tumor microenvironment (TME) between left-sided colon cancer (LCC) and right-sided colon cancer (RCC).

We observed significant differences in TME composition and clinical outcomes between the two groups. Specifically, RCC had a poorer prognosis compared to LCC, particularly in advanced stages (III/IV), consistent with previous findings (4, 33). Bulk RNA sequencing revealed a higher prevalence of immune cells in RCC compared to LCC. Additionally, univariate Cox regression analysis showed that infiltration by specific cell types, such as neutrophils, conventional dendritic cells (cDC), CD4 + memory T cells, resting mast cells, and follicular helper T cells, was linked to better prognosis in colon cancer. Conversely, higher levels of macrophages, naïve CD4 + T cells, and resting natural killer cells were associated with poorer outcomes. Bulk RNA sequencing, while informative, has limitations in accurately representing the distribution of various cell subpopulations within the TME (26, 34). Hence, we utilized single-cell sequencing data to conduct a more comprehensive examination of the tumor microenvironment in the LCC and RCC. Single-cell sequencing analysis revealed distinct variations in major cell clusters composition between LCC and RCC (Figures 2D, E). However, it is important to note that the major cluster analysis only provides a preliminary estimation of cell proportions. To gain a more comprehensive understanding of the tumor microenvironment characteristics and the response to immune checkpoint therapy in LCC and RCC, a more detailed subcluster analysis should be conducted.

Within the tumor microenvironment, our observations indicate that predominant tumor cell subpopulation in RCC tend to exhibited a state of lower differentiation levels of the epithelial tumor cells (Figure 3C) and characterized by a high potential for proliferation and a propensity toward epithelial transition (Figure 3D). These findings are consistent with previous research in this field (35). Notably, tumor cells in RCC exhibit a high expression of major histocompatibility complex class I (MHC I) molecules, whereas tumor cells in LCC exhibited minimal expression (Figure 3D). In patients with colon cancer, those with lower levels of MHC class I expression experienced a significantly worse prognosis compared to those with higher levels (36). MHC class I molecules present peptides derived from self or foreign antigens to CD8 T cells. Therefore, they are essential for antigen specific CD8 T cell immune responses. When cancer cells lose the expression of MHC class I molecules, they can no longer be recognized by conventional CD8 T cells in an antigen specific manner (18). As a result, these cancer cells become resistant to current immunotherapies, including immune checkpoint blockade (e.g., anti-PD-1 therapy) (19). In LCC, despite the presence of immune cell infiltration, tumor-specific cytotoxic T lymphocytes (CTLs) encounter difficulties in exerting their functional role. Additionally, analysis of WES data in colon cancer has revealed widespread gene mutations, including APC, TP53, and KRAS, with mismatch repair serving as the predominant form (Figures 4A, B). These mutations play an important role in tumor proliferation and the transition from epithelial to mesenchymal states. Notably, the frequency of mutations in RCC surpasses that was observed in LCC (Figures 4C, D). Moreover, the elevated frequency of mismatch repair suggested the generation of a greater number of tumor neoantigens, leading to infiltration of tumor-specific CTLs (20). This implies the presence of a greater number of tumor-specific cytotoxic T lymphocytes (CTLs) infiltration in RCC.

Upon analyzing the T cell subsets within the tumor microenvironment, notable distinctions were observed in the composition of T lymphocyte subsets between LCC and RCC. T cells within RCC exhibited a highly differentiated and recently activated state, whereas those within LCC predominantly displayed a low differentiation and naïve state (Figures 5B, G). Within the CD8 positive T-cell populations, cluster C10 expressed exhaustion molecules, coexisting with T cell activation related molecules and tumor killing associated cytokine including IFN-γ, GZMB, TNFRSF9 (Figures 5C, E; Supplementary Table 8), we defined this cluster of cell as tumor-specific CTL, which is in agreement with previous cancer studies (23, 26, 27, 37). The same phenomenon was also observed in the results obtained from flow cytometry and immunohistochemistry (Figures 6A, B). Previous research on phenotypes related to T cell exhaustion has yielded conflicting findings, with certain studies indicating a correlation between T cell exhaustion in the TME and a negative prognosis (38, 39), while others suggest that the presence of T cells expressing exhaustion related molecules is indicative of a positive response from cytotoxic T lymphocytes (37, 40). Consequently, a specific analysis is necessary when categorizing this subset of cells. The increased presence of these cells frequently signifies a positive reaction of the immune system towards the tumor and may result in a more favorable prognosis when utilized in conjunction with immune checkpoint therapy.

Within the CD4 positive T cell populations, exhaustion related molecules are predominantly expressed in the T-reg cell subset, which is associated with immune tolerance (41, 42). By directly inhibiting or indirectly inhibiting anti-tumor immune cells, T-reg cells reduce the effectiveness of anti-tumor immunity. This phenomenon achieved through the secretion of immunosuppressive cytokines like TGF-β and IL-10, as well as through cell-cell contact with other immune cells (43). The elevated expression of this specific subset of cells has been correlated with an unfavorable prognosis (44).

Consequently, when examining the tumor microenvironment, particularly in the context of forecasting the efficacy of immune checkpoint inhibitors in tumor patients, it is imperative to consider multiple factors. These factors encompass the tumor mutational burden, the expression of major histocompatibility complex (MHC) and immune checkpoint molecules, as well as the infiltration of tumor-specific cytotoxic T lymphocytes and regulatory T cells rather than focusing solely on the overall T cell population.

## 5 Conclusion

The tumor microenvironment of right-sided colon cancer (RCC) and left-sided colon cancer (LCC) exhibits distinct characteristics. Specifically, RCC cells show lower levels of epithelial cell differentiation, higher mutational burden, and increased expression of MHC I molecules. Additionally, the tumor microenvironment in RCC is marked by a greater infiltration of tumor-specific cytotoxic T lymphocytes (CTLs). These unique features suggest that RCC patients may benefit more from immune checkpoint inhibitor therapies compared to those with LCC.

## Data Availability

The original contributions presented in the study are included in the article/Supplementary material, further inquiries can be directed to the corresponding author/s.

## Supplementary material

Supplementary Figure 1 The immune landscape of LCC and RCC.

Supplementary Figure 2 The detailed preprocessing of single-cell RNA-seq Data.

Supplementary Figure 3 The gating strategy of flow cytometry.

Supplementary Figure 4 The model plot of the IPS algorithm.

Supplementary Table 1 TCGA and GEO datasets patients information.

Supplementary Table 2 Clinical patients information.

Supplementary Table 3 Key resources information.

Supplementary Table 4 TME score of tumor samples from TCGA and GEO databases.

Supplementary Table 5 Major celltypes counts of COAD scRNA-seq data.

Supplementary Table 6 Major celltypes marker genes.

Supplementary Table 7 Mutation information of LCC and RCC.

Supplementary Table 8 T-cell Subclusters Differential Gene Expression.

Supplementary Table 9 T-cell state markers Genes.

Supplementary Table 10 Flow cytometry Result.
