# Supplementary material for: Multi-omics analysis reveals the landscape of tumor microenvironments in left-sided and right-sided colon cancer
Source: Front Med (Lausanne). 2024 Aug 29;11:1403171. doi: 10.3389/fmed.2024.1403171 (PMC11391487; doi:10.3389/fmed.2024.1403171)
Supplement: Supplementary Figure 1 — The immune landscape of LCC and RCC. [file Data_Sheet_1.pdf]

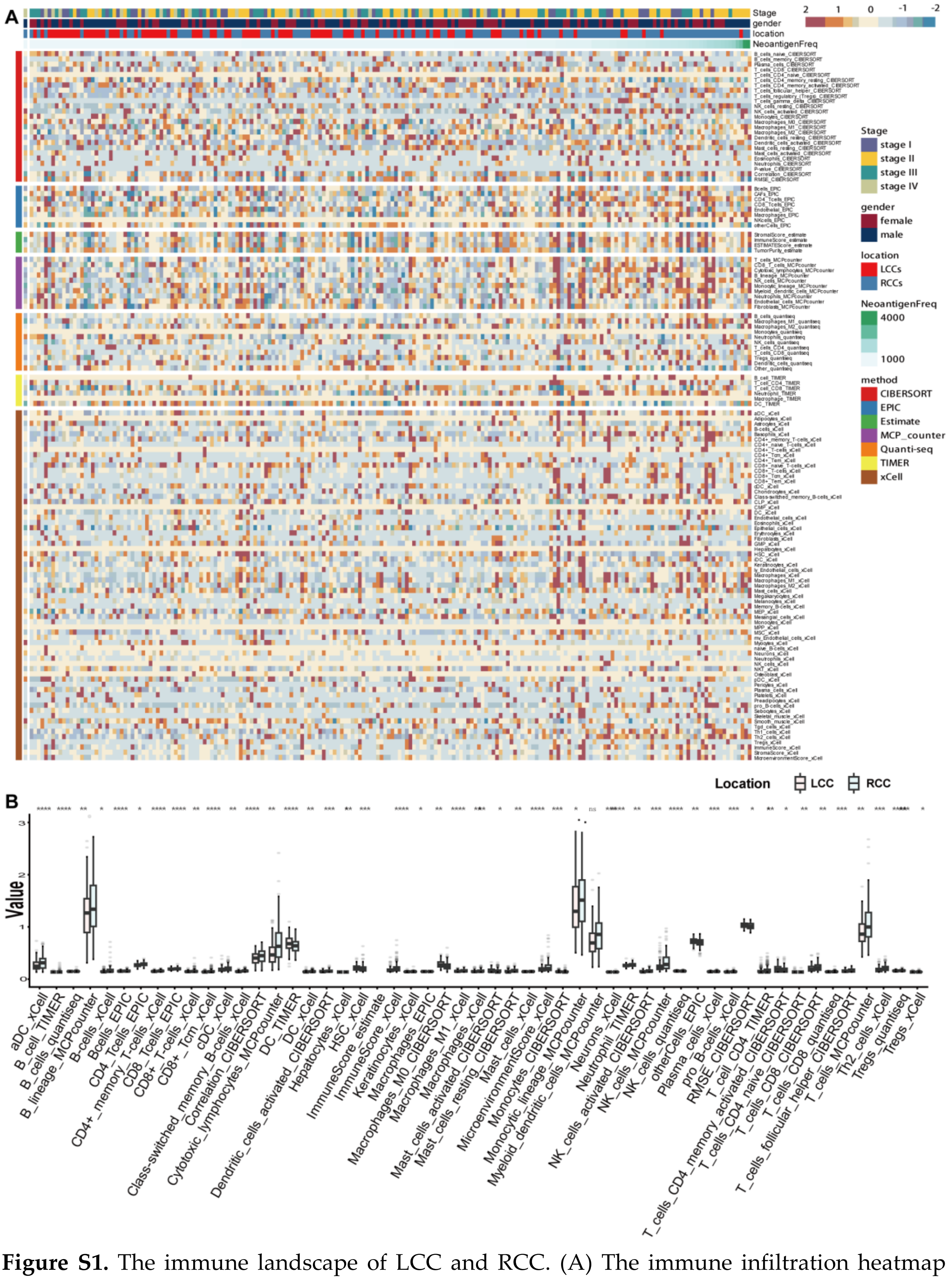

Figure S1. The immune landscape of LCC and RCC. (A) The immune infiltration heatmap between LCC and RCC using deconvolution algorithms. (B) The immune infiltration differences between LCC and RCC, displayed by boxplot. * p<0.05, **p<0.01, ***p<0.001.

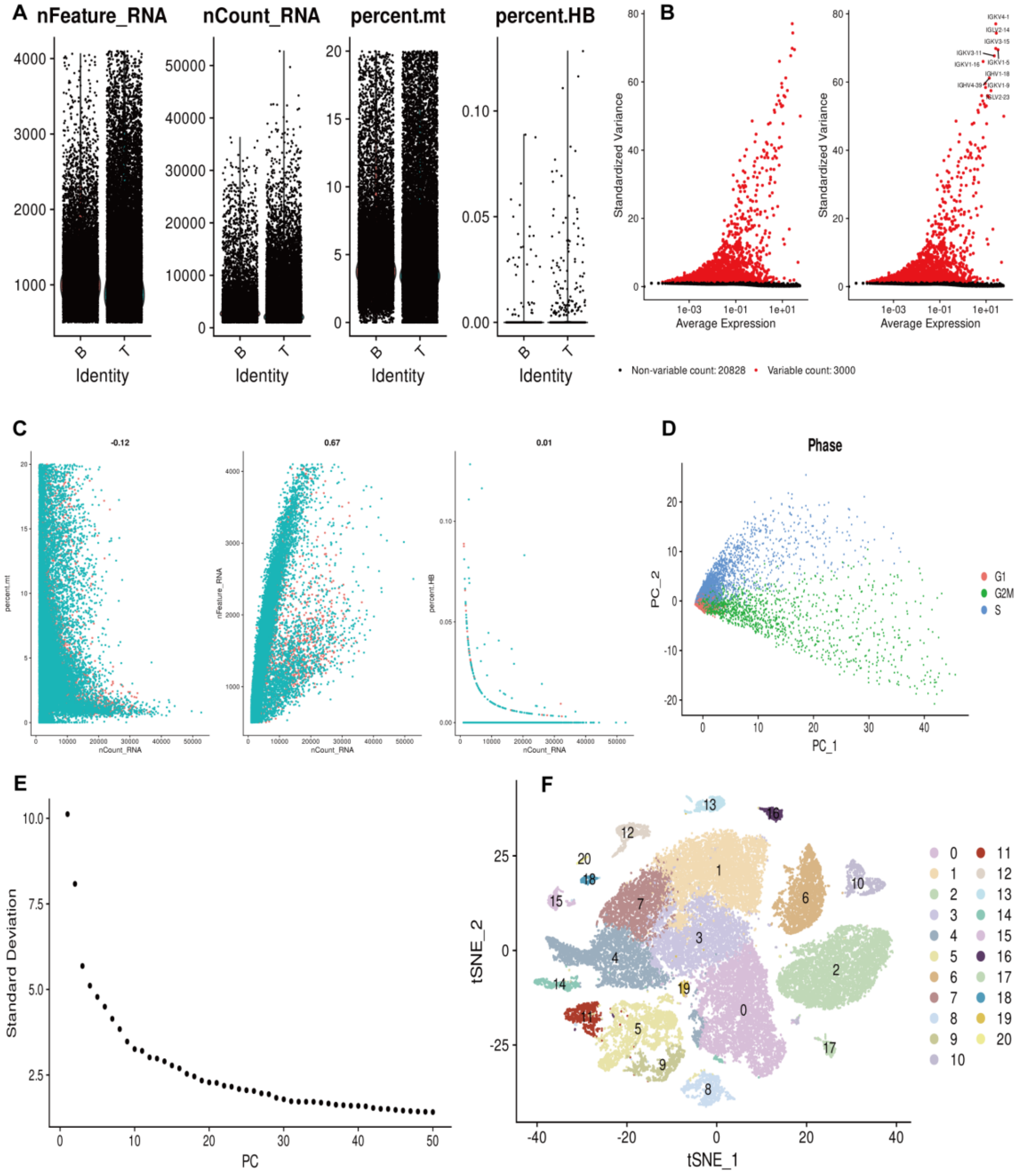

Figure S2. The detailed preprocessing of single-cell RNA-seq Data. (A) After quality control of scRNA-seq data, 42,696 core cells were identified. (B) The variance diagram shows the variation of gene expression in all cells of COAD. The red dots represent highly variable genes and the black dots represent non-variable genes. (C) nCount_RNA, nFeature_RNA and HB_percent were used to visualize the quality control of the single-cell sequencing data.(D) PCA showed a clear separation of cells in COAD. (E) PCA identified the top 20 PCs at $P < 0.05$. (F)The tSNE algorithm was applied to the top 20 PCs for dimensionality reduction, and 21 cell clusters were successfully classified.

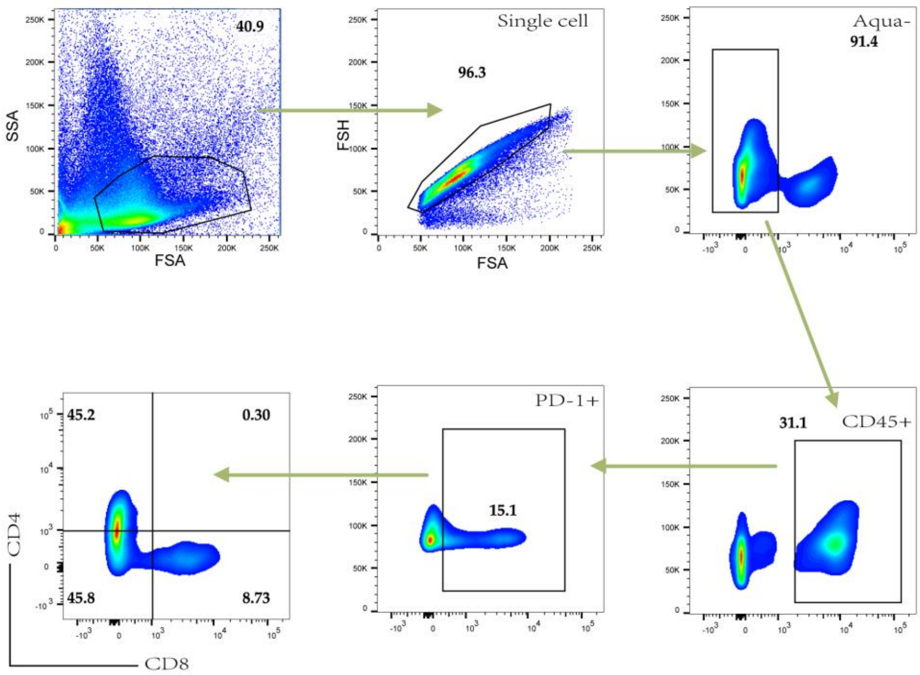

Figure S3. The gating strategy of flow cytometry. In brief, the FSC and SSC dot plots were used to identify live T lymphocytes, and doublets were eliminated with a pulse geometry gate with FSC-H and FSC-A. The T cell viability was further assessed by incubation with Ghost Dye(Aqua). CD45+ immune cell were discriminated from CD45– basal cells.

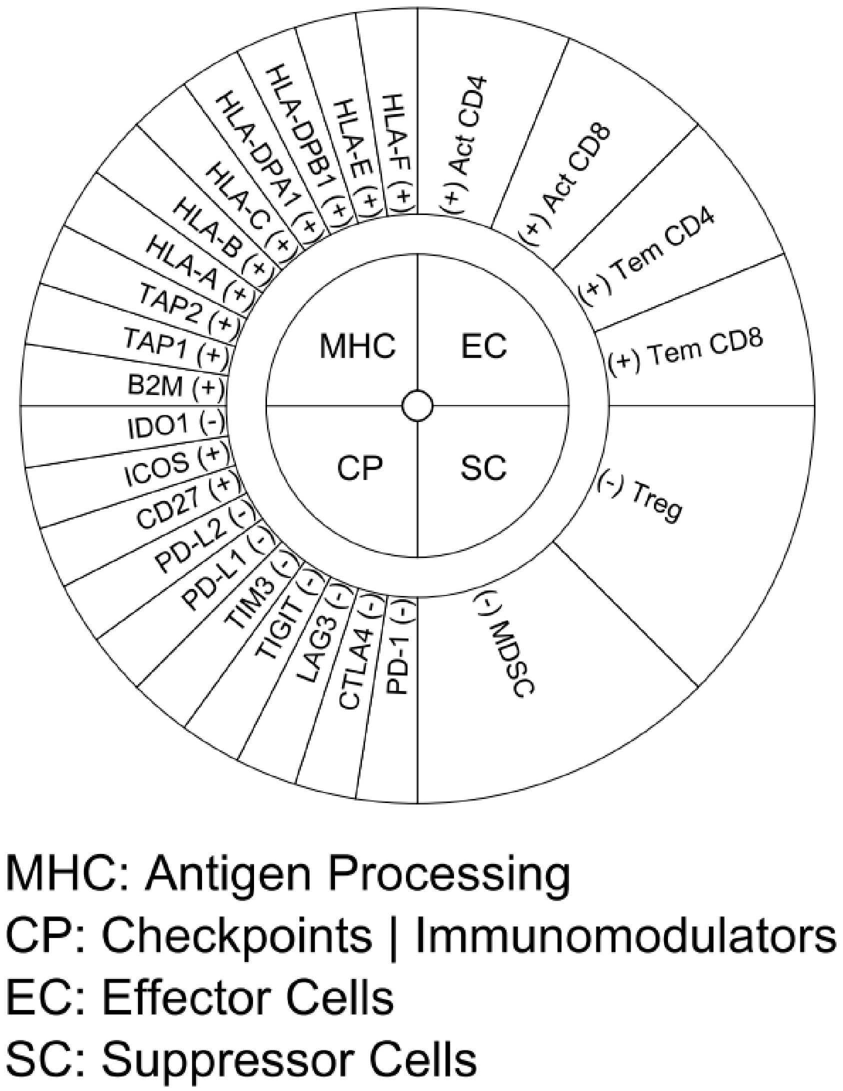

Figure S4. The model plot of the IPS algorithm. The score is based on indicators such as antigen processing, checkpoint immunomodulators, effector cells, suppressor cells, in the bulk RNA-sequencing, to predict the response to immune checkpoint inhibitors.
